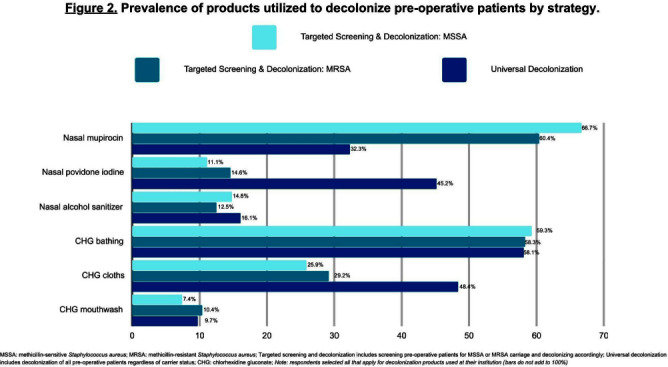# Heterogeneity in Pre-operative Staphylococcus aureus Screening and Decolonization Strategies among Healthcare Institutions

**DOI:** 10.1017/ash.2024.204

**Published:** 2024-09-16

**Authors:** Sarah Bennis, Shalini Kulasingam, Patricia Ferrieri, Susan Kline

**Affiliations:** University of Minnesota

## Abstract

**Background:** Staphylococcus aureus (SA) is the most common pathogen causing surgical site infections (SSIs). In the past decade, strategies incorporating new SA decolonization products have been implemented to prevent SSIs in surgical patients. The objective of this cross-sectional study was to determine which pre-operative screening and decolonization strategies are currently utilized in healthcare institutions. **Methods:** A survey was programmed in REDCap and emailed to members of the Society for Healthcare Epidemiology of America Research Network, the Minnesota chapter of the Association of Practitioners in Infection Control and Epidemiology, and the Minnesota Hospital Association between May-August 2023. We report the prevalence of institutional screening and decolonization strategies and decolonization products used for the prevention of SA SSIs. **Results:** A total of 153 unique institutions initiated the survey and 111 provided complete data on their institutional screening and decolonization strategies. The most commonly reported strategies included universal decolonization (decolonization of pre-operative patients without screening for carrier status) (n=31, 27.9%), no screening or decolonization (n=24, 21.6%), targeted screening for methicillin-sensitive Staphylococcus aureus (MSSA) or methicillin-resistant Staphylococcus aureus (MRSA) and decolonization based on carrier status (n=24, 21.6%), or MRSA only screening and decolonization (n=11, 9.9%) (Figure 1). Institutions that utilized targeted screening and decolonization strategies frequently reported using nasal mupirocin (n=18, 66.7%MSSA, n=29, 60.4%MRSA), chlorhexidine gluconate (CHG) bathing (n=16, 59.3%MSSA, n=28, 58.3%MRSA), and CHG cloths (n=7, 25.9%MSSA, n=14, 29.2%MRSA) (Figure 2). Among the 31 institutions that reported implementing the universal decolonization strategy, CHG bathing (n=18, 58.1%), CHG cloths (n=15, 48.4%), and nasal povidone iodine (n=14, 45.2%) were the most prevalent decolonization products. Additionally, a smaller percentage of institutions used nasal alcohol gel (n=5, 16.1%) for universal decolonization. **Conclusion:** Compared to the survey we conducted in 2012, we report a new shift towards universal decolonization and a small increase in targeted SA screening and decolonization.1 In the 2012 survey we reported 37% of respondents’ institutions screened pre-operative patients for SA carriage and the majority of those institutions decolonized carriers.1 Universal decolonization was not reported in the 2012 survey.1 We highlight the continued heterogeneity in practice at this time, which may reflect the ongoing uncertainty in optimal decolonization practices and emphasizes the need for future research. References: 1. Kline, S. et al. Infect Control Hosp Epidemiol 2014;35(7):880-882.

**Figure:**
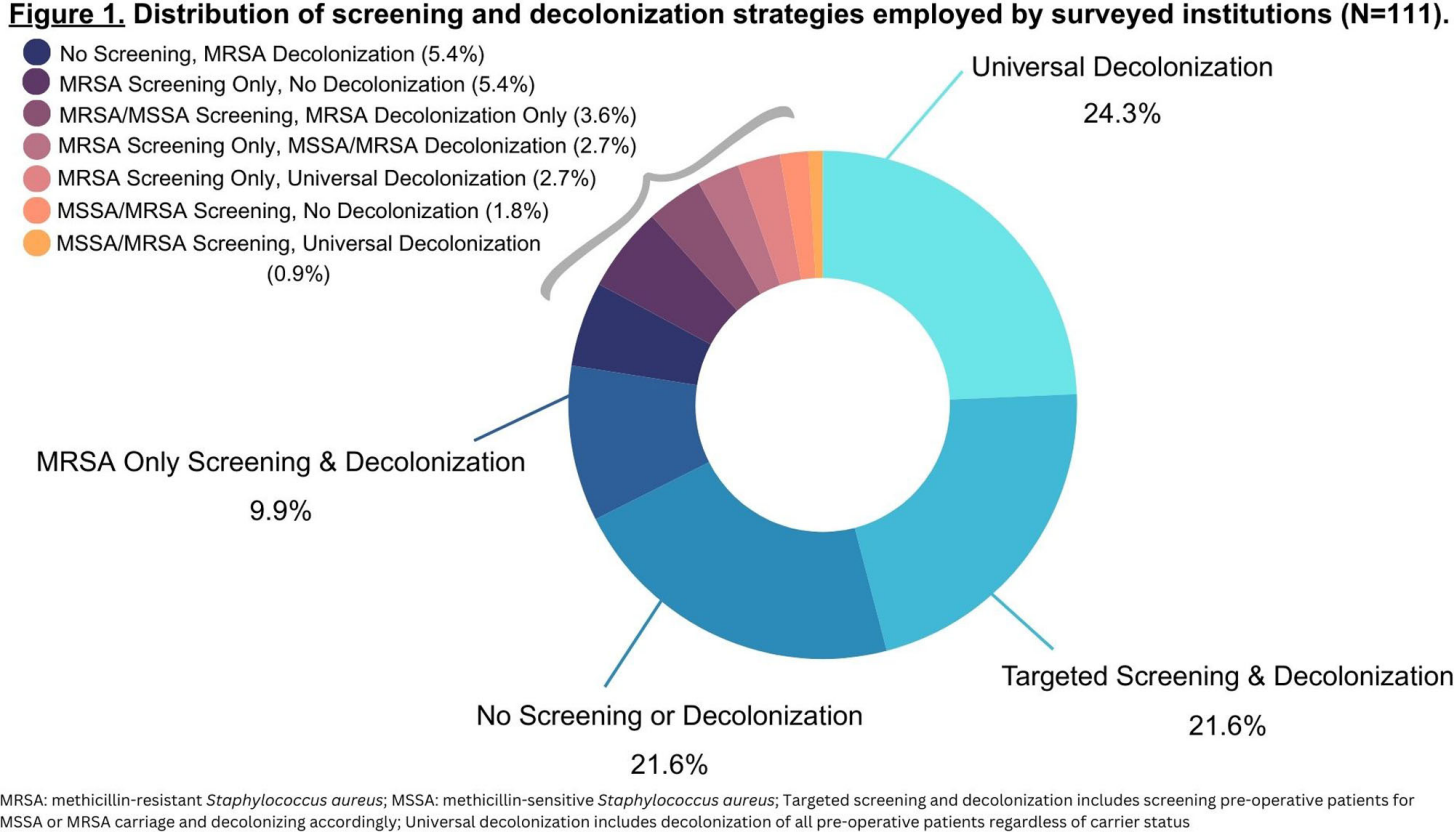

**Figure:**